# Biological Evaluation of Black Chokeberry Extract Free and Embedded in Two Mesoporous Silica-Type Matrices

**DOI:** 10.3390/pharmaceutics12090838

**Published:** 2020-09-01

**Authors:** Valentina Buda, Ana-Maria Brezoiu, Daniela Berger, Ioana Zinuca Pavel, Delia Muntean, Daliana Minda, Cristina Adriana Dehelean, Codruta Soica, Zorita Diaconeasa, Roxana Folescu, Corina Danciu

**Affiliations:** 1 Department of Pharmacology and Clinical Pharmacy, “Victor Babes” University of Medicine and Pharmacy, Eftimie Murgu Square, No. 2, 300041 Timisoara, Romania; buda.valentina.oana@gmail.com; 2 Department of Inorganic Chemistry, Physical-Chemistry & Electrochemistry, Faculty of Applied Chemistry and Materials Science, University “Politehnica” of Bucharest, 1–7 Gheorghe Polizu Street, 011061 Bucharest, Romania; anamariabrezoiu@gmail.com; 3 Department of Pharmacognosy, “Victor Babeş” University of Medicine and Pharmacy, Eftimie Murgu Square, No. 2, 300041 Timisoara, Romania; daliana.minda@umft.ro (D.M.); corina.danciu@umft.ro (C.D.); 4 Department of Microbiology, “Victor Babes” University of Medicine and Pharmacy, Eftimie Murgu Square, No. 2, 300041 Timisoara, Romania; deliacristimuntean@yahoo.com; 5 Department of Toxicology, University of Medicine and Pharmacy “Victor Babeş”, EftimieMurgu Square, No. 2, 300041 Timisoara, Romania; cadehelean@umft.ro; 6 Department of Pharmaceutical Chemistry, University of Medicine and Pharmacy “Victor Babeş”, Eftimie Murgu Square, No. 2, 300041 Timisoara, Romania; codrutasoica@umft.ro; 7 Department of Food Science and Technology, Faculty of Food Science and Technology, University of Agricultural Science and Veterinary Medicine, Calea Manastur, 3–5, 400372 Cluj-Napoca, Romania; zorita.diaconeasa@gmail.com; 8 Department of Anatomy and Embryology, University of Medicine and Pharmacy “Victor Babeş”, Eftimie Murgu Square, No. 2, 300041 Timisoara, Romania; folescu.roxana@umft.ro

**Keywords:** black chokeberry, mesoporous silica, zinc oxide nanoparticles, polyphenols, radical scavenger activity, antimicrobial, A375 human melanoma cell line, HaCaT human keratinocytes

## Abstract

Black chokeberry fruits possess a wide range of biological activities, among which the most important that are frequently mentioned in the literature are their antioxidant, anti-inflammatory, anti-proliferative, and antimicrobial properties. The present paper reports, for the first time, the encapsulation of the ethanolic extract of *Aronia melanocarpa* L. fruits into two mesoporous silica-type matrices (i.e., pristine MCM-41 and MCM-41 silica decorated with zinc oxide nanoparticles). The aim of this work was to evaluate the antiradicalic capacity, the antimicrobial potential, and the effects on the cell viability on a cancer cell line (i.e., A375 human melanoma cell line) versus normal cells (i.e., HaCaT human keratinocytes) of black chokeberry extract loaded on silica-type matrices in comparison to that of the extract alone. The ethanolic polyphenolic extract obtained by conventional extraction was characterized by high-performance liquid chromatography with a photodiode array detector (HPLC–PDA) and spectrophotometric methods. The extract was found to contain high amounts of polyphenols and flavonoids, as well as good radical scavenging activity. The extract-loaded materials were investigated by Fourier transform infrared spectroscopy, N_2_ adsorption–desorption isotherms, thermal analysis, and radical scavenger activity on solid samples. The black chokeberry extract, both free and loaded onto mesoporous silica-type matrices, exhibited a significant antioxidant capacity. Antibacterial activity was recorded only for Gram-positive bacteria, with a more potent antibacterial effect being observed for the extract loaded onto the ZnO-modified MCM-41 silica-type support than for the free extract, probably due to the synergistic effect of the ZnO nanoparticles that decorate the pore walls of silica. The cellular viability test (i.e., MTT assay) showed dose- and time-dependent activity regarding the melanoma cell line. The healthy cells were less affected than the cancer cells, with all tested samples showing good cytocompatibility at doses of up to 100 µg/mL. Improved in vitro antiproliferative and antimigratory (i.e., scratch assay) potential was demonstrated through the loading of black chokeberry extract into mesoporous silica-type matrices, and the screened samples exhibited low selectivity against the tested non-tumor cell line. Based on presented results, one can conclude that mesoporous silica-type matrices are good hosts for black chokeberry extract, increasing its antioxidant, antibacterial (on the screened strains), and in vitro antitumor (on the screened cell line) properties.

## 1. Introduction

The *Aronia* genus (belonging to the Rosaceae family and the Amygdaloideae subfamily) originates from the eastern parts of North America and was initially used by the Native Americans of Forest Potawatomi as tea for the treatment of colds [1,2,3]. Around 1900, it migrated to Europe, initially as ornamental shrubs, and then, around 1946, the plant was settled as a cultivar in Russia. More recently, it spread to East European countries, Germany, Sweden, Norway, and Finland, and, in 1976, it was introduced to Japan [1,2]. *Aronia* fruits are also known as chokeberries, dogberries, choke pears, or wild gooseberries [3]. Its berries are considered an important food ingredient and a source of natural pigments [4], but, due to their astringent properties and sour taste, they are better for processing rather than for direct consumption. Another benefit of *Aronia* cultivation is that it is a relatively easy process, with only occasional ringspot and rust being reported, rather than any major problems (i.e., bird problems, pests, or other serious diseases) [3].

Four species are officially recognized within the genus of *Aronia*: *Aronia melanocarpa* (Michx.) Elliot, known as the black chokeberry; *Aronia prunifolia* (Marshall) Rehder, the purple chokeberry; *Aronia arbutifolia* (L.) Pers, the red chokeberry; and *Aronia mitschurinii* A.K. Skvortsov & Maitul, the dark-fruited chokeberry [1,4].

Nowadays, the cultivated *Aronia melanocarpa* (Michx.) Elliot has a distinct morphology compared to its wild-growing North American counterparts as a result of selection and breeding experiments performed by Ivan Michurin in his nursery at the beginning of the 20th century. It is believed that all black chokeberry plants cultivated in Russia originated from Ivan Michurin’s nursery, the major distinct difference being the large-fruited chokeberry. As reported by Brand et al., it seems that almost all of the scientific investigations found in the literature on *Aronia* fruit content have been performed using *Aronia mitschurinii* (a hybrid containing 75% *A. melanocarpa* and 25% *Sorbus aucuparia*, and which is the only species used for commercial fruit production), and not on *A. melanocarpa* (Michx.) Elliot due to an inadequacy in understanding the taxonomy and genetics of *Aronia* [1,5].

Black chokeberry (*A. melanocarpa* (Michx.) Elliott) fruits have been reported to be an enormous source of bioactive compounds, such as polyphenols (anthocyanins and procyanidins in particular), other phenolic compounds such as chlorogenic and neo-chlorogenic acid and small amounts of tannins, pectin, a low amount of fat (mainly phosphatidylinositol and glycerides from linoleic acid), mineral compounds (e.g., K, Zn, Na, Ca, Fe, and Mg), vitamins (i.e., C, B1, B2, B6, niacin, folic acid, pantothenic acid, carotenoids, and α- and β-tocopherol), triterpenes (mainly β-sitosterol and campesterol), carbohydrates, amygdalin, and over 40 volatile compounds [2,3,4]. Derivatives of betulinic acid, namely, 23-hydroxybetulinic acid and 2α-hydroxyoleanoic acid, were found in the seedlings of black chokeberry [3]. Several studies have emphasized that the concentration of bioactive compounds depends on the cultivar, habitat, fertilization, maturation of berries, and harvest date [2].

Several in vitro and in vivo studies have highlighted the antioxidant (one of the richest plants in antioxidants), anti-inflammatory, anti-proliferative, anti-atherosclerotic, hypotensive, antiplatelet, cardioprotective, gastroprotective, antidiabetic, antimicrobial (antibacterial and antiviral), immunomodulatory, and antitumor potential of the fruits, the juice, or the different types of concentrated or standardized extracts [1,4].

Mesoporous silica nanoparticles (MSNs) have gained extremely high consideration and importance in recent years, as they can be used as matrices for many pharmacologically active compounds based on their versatile properties not only in drug delivery, but also in imaging applications [6]. Moreover, in 2011, they were approved by the Food and Drug Administration for phase I clinical trials in humans. Mobil crystalline material (MCM-41), together with SBA-15, are the most studied silica-type carriers, which present two-dimensional (2D) hexagonal mesopore channels [7]. They operate as matrices for biologically active molecules, having several relevant properties, such as a high surface area, large pore volume, high capacity to accommodate guest molecules, and good mechanical and thermal stability. They also possess good biodegradability and can be used to enhance the solubility and dissolution rate of poor water-soluble drugs due to their amorphization when adsorbed into mesoporous MSNs, and, thus, to improve their bioavailability. Based on their size, they can easily accumulate at the needed site of action through an increased permeability and enhanced retention effect. Based on their shape, they are rapidly internalized by cells and tissues. As a consequence, MSNs have been found to be good hosts for drugs, not only in cancer treatment [8,9,10,11,12] but also in anti-biotherapy as they can improve antibiotic release kinetics, thus increasing patients’ adherence and decreasing the risk of developing resistance [7,13,14,15,16].

However, regarding the employment of MSNs for embedding polyphenolic extracts, there are only a few reports [17,18,19] in which it is mentioned that, through encapsulation, improved stability can be achieved in comparison to that of the extract alone, which is prone to a faster degradation. Evidence of the benefits of phytochemical encapsulation has also been found by using a bio-hybrid matrix consisting of *Althaea officinalis* L. (i.e., polysaccharides) and *Betonica officinalis* L. (i.e., polyphenols) extract-loaded SBA-15-type silica, showing chemopreventive effects against Hep-2 human epidermoid carcinoma of larynx cell lines and non-toxicity against NCTC clone 929 mouse fibroblast cells [20].

Recently, it was reported that encapsulation of black chokeberry extract in maltodextrin and skimmed milk by spray drying [21] or in alginate and alginate–inulin systems by electrostatic extrusion [22] leads to the increased stability of phytochemicals [23]. In addition, the encapsulation of *Aronia* juice in maltodextrin and Arabic gum has been shown to preserve anthocyanin content [24].

This paper reports, for the first time, the encapsulation of black chokeberry extract into MCM-41-type mesoporous silica (pristine and modified with zinc oxide nanoparticles). The aim of this study was to evaluate the activity of two different formulations containing encapsulated *A. melanocarpa* polyphenolic extract in comparison to that of the free extract based on their preliminary biological tests, such as their antiradical and antioxidant capacity, antimicrobial potential on the selected strains, and in vitro effects on cancer cell viability (using A375 human melanoma cells) and normal skin cells (using HaCaT human keratinocytes).

## 2. Materials and Methods

### 2.1. Materials

For the synthesis of mesoporous silica, tetraethyl orthosilicate (TEOS; Fluka, Seelzer, Germany), trimethylhexadecylammonium bromide (CTAB; Alfa Aesar, Ward Hill, MA, USA), zinc acetate dihydrate (Zn(CH_3_COO)_2_·2H_2_O; Fluka, Seelzer, Germany), NH_4_Cl (Sigma-Aldrich Co. Merck Group, Darmstadt, Germany), 36.5–38% wt hydrochloric acid (Merck Group, Darmstadt, Germany), and 25% wt ammonia aqueous solution (Scharlau, Scharlab S.L., Barcelona, Spain) were used as received without further purification.

The reagents used in the spectrophotometric determinations were sodium carbonate (Na_2_CO_3_), potassium persulfate (K_2_S_2_O_8_), aluminum chloride hexahydrate (AlCl_3_·6H_2_O), Folin–Ciocalteu reagent, 2,2′-azino-bis(3-ethylbenzothiazoline-6-sulphonic acid) (ABTS), and 2,2-diphenyl-1-picrylhydrazyl (DPPH), purchased from Sigma-Aldrich Co. (Merck Group, Darmstadt, Germany), as well as 6-hydroxy-2,5,7,8-tetramethylchroman-2-carboxylic acid (Trolox, 97%; Aldrich Chemical Co., Inc., Milwaukee, WI, USA). Ascorbic acid powder was bought from a local vendor.

For the chromatographic analyses, several standard HPLC-grade compounds were used: gallic acid purchased from Alfa Aesar (Ward Hill, MA, USA); vanillic, protocatechuic, *trans*-ferulic, and chicoric acids, (−)epicatechin, and ellagic acid dihydrate from TCI (Tokyo, Japan); caftaric and syringic acid purchased from Molekula GmbH (Munich, Germany); chlorogenic acid from HWI group (Alpen Aan de Rijn, The Netherlands); rosmarinic and caffeic acid, quercetin, rutin and catechin hydrate, myricetin, kaempferol, and cyanidin chloride acquired from Sigma-Aldrich Co. (Merck Group, Darmstadt, Germany); *trans-p*-coumaric acid, *trans*-resveratrol, malvidin chloride, and delphinidin chloride from Sigma-Aldrich Co. (Merck Group, Darmstadt, Germany); pelargonidin chloride bought from Aldrich Chemical Co., Inc. (Milwaukee, WI, USA); solvents such as ethanol and acetonitrile (ACN) purchased from Riedel-de Haën (Honeywell Riedel-de Haën, Seelzer, Germany); and formic acid from Merck Group (Darmstadt, Germany) used without additional purification. For all solutions and experiments, ultrapure water (Millipore Direct-Q 3 UV water purification system with a Biopack UF cartridge) was used.

### 2.2. Preparation and Characterization of the Black Chokeberry Polyphenolic Extract

The ethanolic polyphenolic extract from *A. melanocarpa* (Michx.) Elliott fruits was prepared using a finely ground powder of *Aronia* dried berries (from the western part of Romania) and ethanol by conventional extraction. The *Aronia* dried berries (recognized in the Department of Pharmacognosy, “Victor Babes” University of Medicine and Pharmacy Timisoara; a voucher specimen (No. AM_AM1) was deposited at the Herbarium of the Faculty of Pharmacy, Timisoara) were macerated (*Aronia* dried berries /ethanol = 1/6 *w*/*v*) at room temperature overnight and were then heated at reflux in three 1-h stages, with magnetic stirring, intermediate filtration, and solvent replacement at the same ratio as in the maceration process. Finally, the extract fractions were combined and dried under vacuum and further re-dissolved in ethanol.

The *A. melanocarpa* polyphenolic extract was characterized using spectrophotometric methods (Shimadzu UV-1800, Shimadzu Corporation, Kyoto, Japan) to determine the total polyphenolic (using Folin–Ciocalteu assay, in the 50–450 μg/mL gallic acid domain and expressed as milligrams of gallic acid equivalent (GAE) per gram of dried black chokeberry), flavonoid (AlCl_3_ assay, expressed as rutin hydrate equivalent (RE) in the 0.5–100 μg/mL rutin hydrate domain), and anthocyanin (using the extinction coefficient of cyanidin-3-glicoside and expressed as cyanidin-3-glucoside equivalent (CGE)) contents, and radical scavenger activity (RSA) was determined by the in vitro DPPH method (50 µL extract or standard substance mixed with 2.95 mL of 25 mg/L DPPH ethanolic solution) and the ABTS assay (20 µL extract or standard substance mixed with 980 µL ABTS carbocation radical solution, generated by the reaction of 10 mL of 7 mM aqueous solution of ABTS and 176 µL of 2.45 mM aqueous solution of K_2_S_2_O_8_), expressed as Trolox equivalent (TE) in the 0.05–1.25 mM domain. The extract composition was determined by reversed-phase high-performance liquid chromatography with a photodiode array detector (HPLC–PDA) (Shimadzu Nexera 2, Shimadzu Corporation) as previously described [17]. The limit of detection (LOD) for the standard substances identified in the black chokeberry extract was 0.099 mg/L for protocatechuic, chlorogenic, and caffeic acids or 0.499 mg/L for rutin hydrate, while the limit of quantification (LOQ) was 0.494 mg/L (protocatechuic acid), 0.495 mg/L (chlorogenic and caffeic acids), or 0.998 mg/L (rutin hydrate).

### 2.3. Encapsulation of the Black Chokeberry Extract into Mesoporous Silica-Type Supports

As supports for polyphenolic extract loading, pristine MCM-41 silica (MCM-41E) and MCM-41 silica with pore walls decorated with ZnO nanoparticles (ZnMCM-41) were used. The synthesis procedure of these supports was previously reported [25]. The materials containing black chokeberry extract were prepared by the incipient wetness impregnation method using *A. melanocarpa* ethanolic extract and MCM-41E or ZnMCM-41 supports. The polyphenolic extract was mixed with the silica-type supports, previously outgassed at 110 °C for 18 h, using the outgassing unit of a Quantachrome Autosorb iQ_2_ gas sorption analyzer (Quantachrome Instruments, Boynton Beach, FL, USA), and the resulted suspension was dried under vacuum for 12 h in static conditions, at room temperature, at 4 mbar, in a desiccator connected to a vacuum diaphragm pump (Vacuubrand diaphragm pump MD 1, Wertheim, Germany). The extract-loaded materials were labeled Ar@support.

### 2.4. Material Characterization

The mesoporous carriers were characterized using Fourier transform infrared spectroscopy (FTIR), nitrogen adsorption–desorption isotherms, and scanning electron microscopy coupled with energy dispersive X-ray spectroscopy (SEM-EDS), while the extract-loaded materials were investigated by FTIR spectroscopy, N_2_ adsorption–desorption isotherms, thermogravimetric analysis (TGA)/differential thermal analysis (DTA), and RSA on solid samples.

The FTIR spectra were recorded in the 4000–400 cm^−1^ range on a Bruker Tensor 27 spectrophotometer (KBr pellet technique; Bruker Corporation Optik GmbH, Bremen, Germany). Nitrogen adsorption–desorption isotherms were recorded at 77 K using a Quantachrome Autosorb iQ_2_ gas sorption analyzer. The specific surface area values, S_BET_, were computed through the Brunauer–Emmett–Teller method in the relative pressure range of 0.05–0.25, while the pore size distribution curves and the average pore diameter, *d*_BJH,_ were determined based on the Barrett–Joyner–Halenda (BJH) method and the total pore volume was determined at *p*/*p*_0_ = 0.99. Prior to recording the isotherms, the mesoporous supports and extract-loaded samples were outgassed at 110 °C for 12 h and 35 °C for 18 h, respectively.

The chemical composition of the ZnMCM-41 support was determined by SEM-EDS (Tescan Vega 3 LMH, Brno, Czech Republic and Bruker Nano, Berlin, Germany), while the phytochemical content for the extract-loaded samples was evaluated through TGA/DTA performed on a Mettler Toledo GA/SDTA851e equipment (Greifensee, Switzerland) in the temperature range of 25–600 °C in air, at a heating rate of 10 °C/min.

The RSA of the extract-loaded materials was assessed by DPPH assay; the details of this method applied to solid samples were reported elsewhere [17]. In brief, the extract-loaded powders were analyzed in comparison to the corresponding carrier, as well as the free extract in the same concentrations, with the degradation of the DPPH free radical solution as the control, after 24 h.

### 2.5. Methodology

#### 2.5.1. In Vitro Antimicrobial Activity

The compounds were screened for their antimicrobial activity against 10 reference strains (Thermo Fisher Scientific, Waltham, MA, USA), as shown in Table 1.

We selected these 10 strains because they are the most common pathogens (i.e., bacteria and fungi) responsible for healthcare-associated infections and because they cause continuous problems for the healthcare industry (i.e., high medical costs and treatment resistance); thus, they can be seen as economic burdens.

The antimicrobial activity of the samples was evaluated by both the agar disk diffusion method and the dilution method, according to the procedures outlined by the Clinical Laboratory and Standard Institute (CLSI), the European Committee on Antimicrobial Susceptibility Testing (EUCAST), and the other studies [26,27,28,29,30].

##### Disk Diffusion Method

For each microorganism under study, suspensions were prepared in physiological saline to a 0.5 McFarland standard concentration. Mueller–Hinton agar (Sanimed, Bucharest, Romania), supplemented with 5% defibrinated sheep blood for *Streptococcus* spp., was inoculated with 0.1 mL of this suspension. Ten microliters from each sample (8 mg dry substance/1 mL ethanol) were added to a 6-mm diameter blank disk (BioMaxima, Lublin, Poland), placed on top of the Mueller–Hinton medium. The plates were incubated at 35–37 °C for 24 h, and readings of the inhibition zones were then taken in millimeters. All tests were done in triplicate and the average value was recorded. The positive control consisted of a gentamycin and fluconazole disk (BioMaxima, Lublin, Poland), while a blank paper disk impregnated with ethanol was used as a negative control.

##### Dilution Method

An inoculum was prepared from the 0.5 McFarland microbial suspension by dilution of 1:150 in order to obtain a suspension of 10^6^ colony-forming units (CFU)/mL. A further dilution of 1:2 brought the final inoculum to 5 × 10^5^ CFU/mL. For the tested compounds, serial dilutions of 8, 4, 2, 1, 0.5, and 0.25 mg /mL were prepared. In six test tubes, 0.1 mL of each dilution of the tested compound, 0.4 mL of the Mueller–Hinton broth (supplemented with 2–5% lysed horse blood for *Streptococcus* spp.), and 0.5 mL of the suspension of the microorganism under study (final inoculum was approximately 0.5 × 10^5^ CFU/mL) were added. After incubation at 37 °C for 24 h, the minimum inhibitory concentration (MIC) (i.e., the lowest concentration without visible growth) was determined. As a control, 0.1 mL of ethanol was added into a tube with 0.5 mL of the microbial suspension and 0.4 mL of the Mueller–Hinton broth.

To determine the minimum bactericidal concentration (MBC), a volume of 1 µL from the test tubes with no visible growth was inoculated using a loop (NuovaAptaca SRL, Italy) on Columbia agar supplemented with 5% blood. The inoculated plates were incubated at 37 °C for 24 h. To determine the minimum fungicidal concentration (MFC), a Sabouraud with chloramphenicol medium was used for inoculation.

#### 2.5.2. In Vitro Cytotoxic Activity

##### Cell Culture

The human melanoma cell line A375 (ATCC^®^ CRL-1619^TM^) was acquired from the American Type Culture Collection (ATCC, Manassas, VA, USA) and the HaCaT human keratinocytes were kindly given by the University of Debrecen (Debrecen, Hungary). The cells were cultured in high-glucose Dulbecco’s Modified Eagle’s Medium (DMEM; Sigma-Aldrich, Taufkirchen, Germany) supplemented with 1% antibiotic mixture (Penicillin/Streptomycin (Pen/Strep), 10,000 IU/mL; Sigma-Aldrich, Taufkirchen, Germany) in order to avoid contamination and 10% fetal bovine serum (FBS; Gibco, Thermo Fisher Scientific, Waltham, MA, USA). Standard conditions (37 °C and humidified atmosphere containing 5% CO_2_) were used for the cell culture.

##### MTT Assay

The MTT technique was used to determine cellular viability. For this, 1 × 10^4^ cells/well were seeded in 96-well microplates and allowed to attach overnight. Then, the cells were treated with different concentrations (i.e., 10, 50, 100, and 250 µg/mL) of the samples (i.e., *A. melanocarpa* extract, Ar@MCM-41E, Ar@ZnMCM-41, and MCM-41E and ZnMCM-41 matrices) and incubated for 24, 48, or 72 h. The control group is represented by untreated cells, incubated only with cell culture medium. After the treatment period, 10 μL of 5 mg/mL MTT (3-(4,5-dimethylthiazol-2-yl)-2,5-diphenyltetrazolium bromide) solution (Sigma-Aldrich, Taufkirchen, Germany) were added into each well. The cells were incubated for 3 h at 37 °C; during this period of time, the intact mitochondrial reductase converted and precipitated MTT as blue crystals. A volume of 100 μL of lysis solution was added in each well to dissolve the precipitated crystals. Then, the absorbance was spectrophotometrically measured at 570 nm using a microplate reader (xMark Microplate Spectrophotometer, Bio-Rad, Serial No. 10578, Tokyo, Japan).

##### Scratch Assay

This method was used to evaluate the antimigratory potential of the samples (i.e., *A. melanocarpa* extract, Ar@MCM-41E, Ar@ZnMCM-41, and MCM-41E and ZnMCM-41 matrices) against the A375 human melanoma cell line. First, 2 × 10^5^ cells/well were cultured in 12-well plates until a confluence of 90% was reached. After that, scratches were drawn on well-defined zones of the cells’ monolayer with a sterile pipette tip. Cells that detached after the procedure were removed by washing with phosphate-buffered saline (PBS) before treatment with the samples. Then, the cells were treated with a concentration of 100 µg/mL from each sample. Pictures of the cells were taken at 0 and 24 h using an Olympus IX73 inverted microscope provided with DP74 camera (Olympus, Japan). For comparation, we used a control group (i.e., untreated cells). For the analysis of cell migration, we used cell Sense Dimension Software. To determine the migratory ability of the tumor cells, the scratch closure rate was calculated as previously described [31]:

(1) $$\mathrm{Scratch}\;\mathrm{closure}\;\mathrm{rate}\;=\;\left[\frac{A_{to}-A_{t}}{A_{to}}\right]\times 100$$

where *A_t_*_0_ is the scratch area at 0 h and *A_t_* is the scratch area at 24 h.

#### 2.5.3. Statistical Analysis

For the in vitro analysis, the results are expressed as mean ± standard deviation (SD). Comparison among groups was performed by one-way analysis of variance (ANOVA), followed by a Dunnett’s multiple comparison test. GraphPad Prism 5 (GraphPad Software, San Diego, CA, USA) was used for the statistical analysis.

## 3. Results

### 3.1. Obtaining and Characterizing the Black Chokeberry Polyphenolic Extract

The *Aronia* ethanolic extract had high concentration of polyphenols, flavonoids, and anthocyanins, as well as good radical scavenging activity (Table 2). All group quantification methods are expressed per gram of dried berries, with a total polyphenol content of 17.32 ± 0.18 mg GAE/g, a total flavonoid content of 6.98 ± 0.28 mg RE/g, and a total anthocyanin content of 0.52 ± 0.02 mg CGE/g. The RSA, expressed as TE, was in the range of 14.06–15.33 mg TE/g, with a slightly higher value being obtained from the ABTS assay.

**Figure 1:**
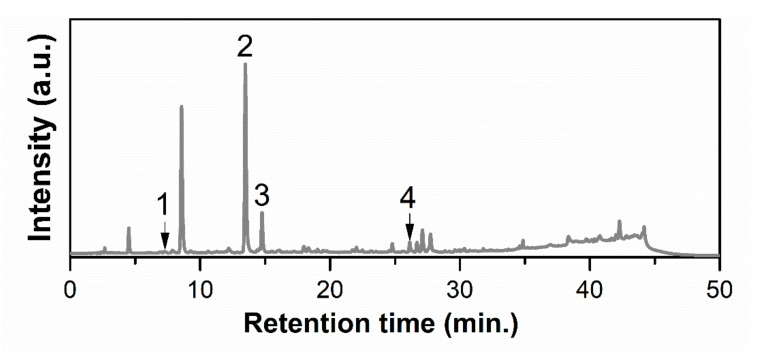
HPLC–PDA chromatogram of the *Aronia* extract at 326 nm (1, protocatechuic acid; 2, chlorogenic acid; 3, caffeic acid; 4, rutin hydrate).

HPLC–PDA analysis led to the identification of four compounds from the 23 available standards, and the corresponding chromatogram is presented in Figure 1. The most abundant substance was chlorogenic acid (141.283 ± 0.045 mg/100 g), followed by protocatechuic acid (62.135 ± 0.005 mg/100 g) and rutin hydrate (21.773 ± 0.041 mg/100 g), with caffeic acid being found in the lowest amount (0.725 ± 0.008 mg/100 g).

### 3.2. Characterization of Aronia-Loaded Mesoporous Silica-Type Supports

The mesoporous silica-type materials used for embedding the *Aronia* extract, MCM-41E, and ZnMCM-41 (12% wt ZnO determined by EDS) were chosen due to their high pore volume, in the range of 0.78–0.80 cm^3^/g (Table 3). The *Aronia* extract-loaded materials were characterized by FTIR spectra, TGA/DTA, and N_2_ adsorption–desorption isotherms.

**Figure 2:**
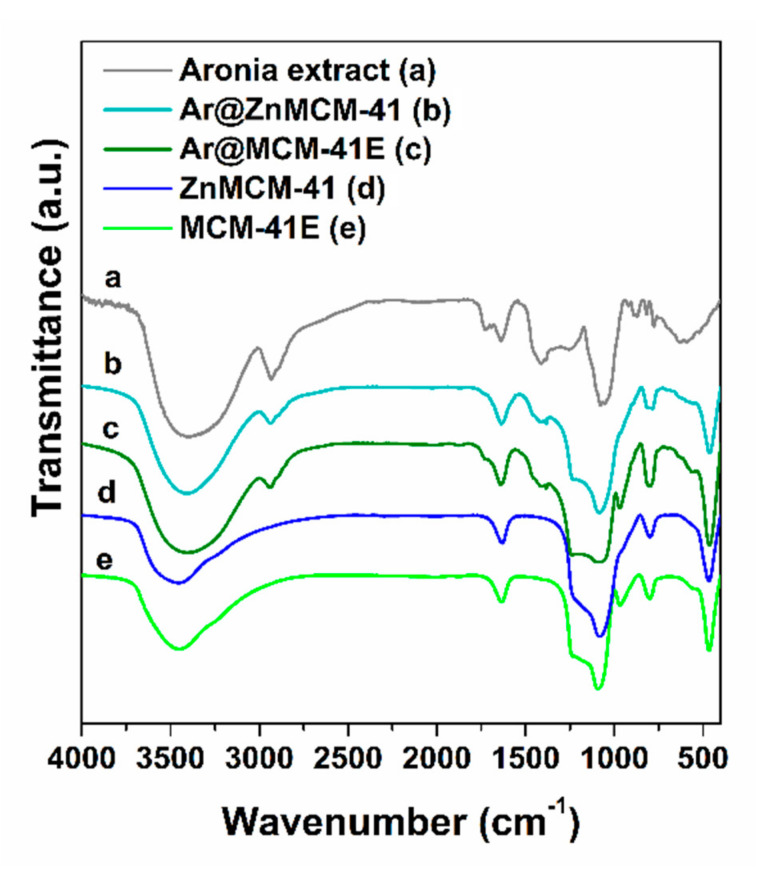
FTIR spectra of the *Aronia* extract (**a**) and extract-loaded materials (**b**,**c**) in comparison to the corresponding supports (**d**,**e**).

**Figure 3:**
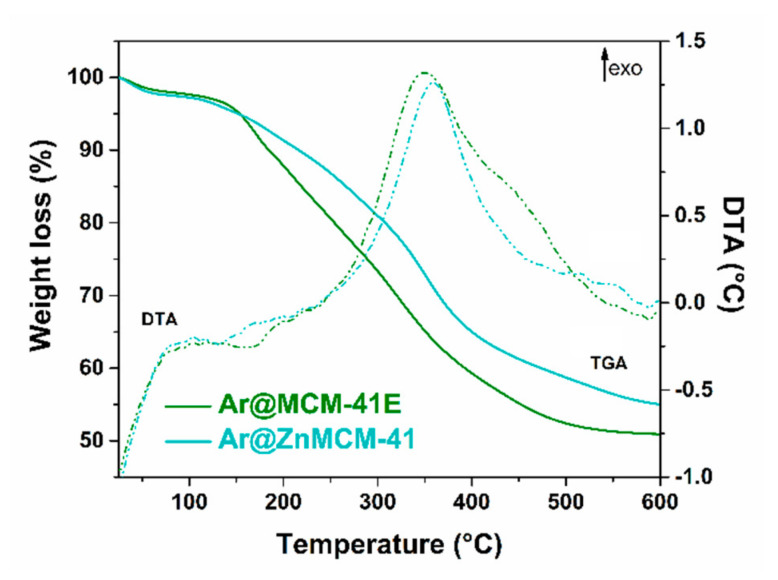
TGA/DTA of the extract-loaded silica-type supports.

The FTIR spectra of the *Aronia* extract-loaded samples evidenced the presence of phytocompounds on the mesoporous silica-type supports through the bands associated with the stretching vibrations of C–H bonds (in the 2850–3000 cm^−1^ region) and C–O bonds (1732 cm^−1^ for the embedded extract and 1726 cm^−1^ for the polyphenolic extract). The bands assigned to the mesoporous silica-type supports were asymmetrical and symmetrical stretching vibrations of Si–O–Si bonds (1088 and 804 cm^−1^ for the MCM-41E support and 1084 and 798 cm^−1^ for ZnMCM-41, respectively), stretching vibration of silanol groups at 968 cm^−1^, and the deformation band of Si–O bonds at 468 cm^−1^, as well as the specific band of physiosorbed water molecules at 1638 cm^−1^ (Figure 2). The content of natural compounds in the extract-loaded samples, which was in the range of 42–43% wt (Table 3), was determined from the thermogravimetric analysis considering the weight loss up to 600 °C and after deducting the physiosorbed water associated with the first endothermic event on the DTA curve (Figure 3).

**Figure 4:**
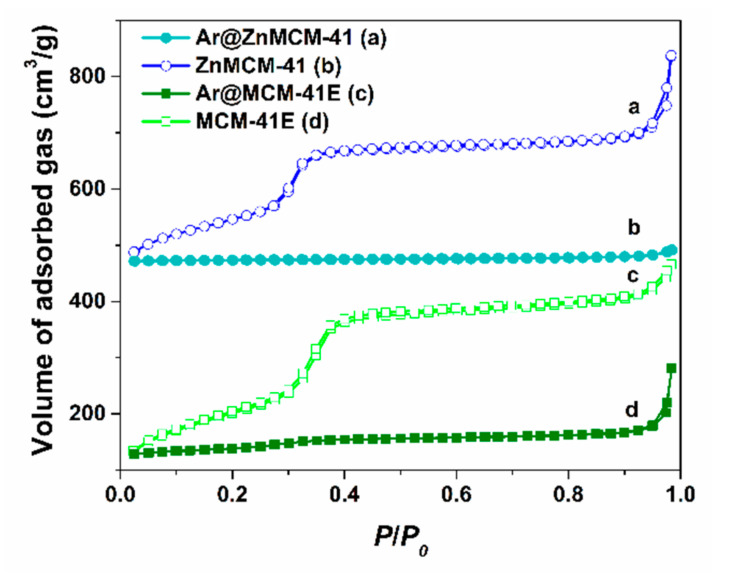
Nitrogen adsorption–desorption isotherms of Ar@ZnMCM-41 (**b**) and Ar@MCM-41E (**d**) in comparison to the corresponding ZnMCM-41 (**a**) and MCM-41E (**c**), respectively.

The textural parameters of the supports and the extract-loaded materials determined from the N_2_ adsorption–desorption isotherms (Figure 4) are listed in Table 3. One can observe a sharp decrease in porosity as result of the adsorption of natural compounds into the mesopores of the silica-type supports. Hence, the specific surface area computed with the Brunauer–Emmett–Teller method, *S*_BET_, decreased from 781 and 620 m^2^/g to 89 and 17 m^2^/g for MCM-41E and ZnMCM-41, respectively, and the total pore volume, *V*_pore_, from 0.78 and 0.80 cm^3^/g to 0.12 and 0.11 cm^3^/g for MCM-41E and ZnMCM-41, respectively. The adsorption of phytochemicals from *Aronia* into the pores of the silica-type supports was also confirmed by a decrease in the average pore size, *d*_BJH_, from 2.81 nm for MCM-41E to 2.52 nm for Ar@MCM-41E.

**Figure 5:**
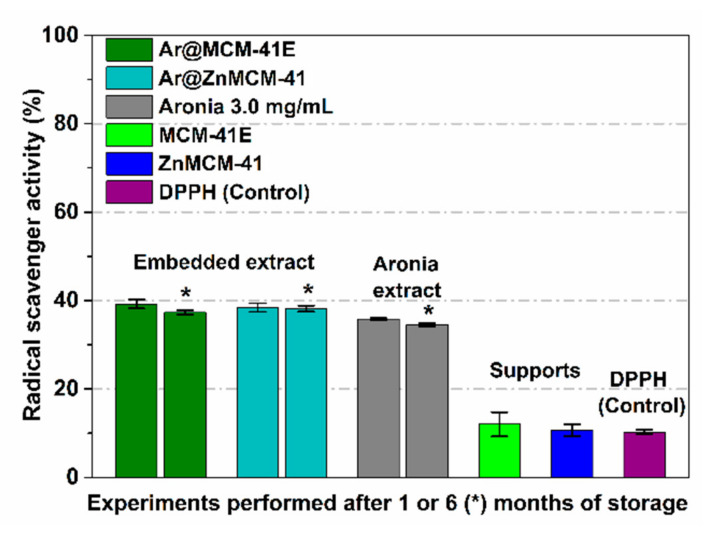
In vitro RSA for the extract-loaded supports in comparison to that of the corresponding support and free extract after one and six months (*) of storage.

The RSA of black chokeberry-loaded materials was determined after 24 h of incubation in DPPH free radical solution under dark conditions, in duplicate, and compared to that of the free extract and the corresponding silica-type support in the same concentration as in the materials containing the extract, using the degradation of the DPPH free radical solution as the control. Considering that mesoporous silica-type supports have the ability to adsorb organic molecules (i.e., DPPH radicals) due to their high porosity, as well as the slow interaction between free radicals from the solution and a solid sample (antioxidant compounds encapsulated in a solid matrix), 24 h of incubation was determined as an optimum duration in the case of the embedded extract in a solid support [17]. In addition, the degradation of the DPPH solution over time was considered for the determination of the RSA for all samples. One can observe the preservation of RSA for the embedded extract, which was slightly higher than that of the free extract (Figure 5), while the mesoporous silica-type supports did not contribute to the RSA.

### 3.3. Antimicrobial Activity

Table 4 presents the antimicrobial activity of the black chokeberry extract free and embedded in two mesoporous silica-type matrices (i.e., Ar@MCM-41E and Ar@ZnMCM-41), which was assessed by two methods: the disk diffusion method (expressed through the dimeter of the inhibition zone) and the dilution method (expressed through the MIC and the MBC/MFC).

The free black chokeberry extract was active only in the Gram-positive cocci strains, with the best antibacterial effect on *Streptococcus pyogenes* ATCC 19615 strain (inhibition zone diameter: 16.66; MIC equivalent to an MBC of 1 mg/mL), followed by the *Streptococcus pneumoniae* ATCC 49619 strain and the *Staphylococcus aureus* ATCC 25923 strain (both with an MIC and an MBC of 2 mg/mL).

The two formulations of black chokeberry extract embedded in the mesoporous silica-type matrices were more effective. It can be noticed that the best antibacterial effect of the Ar@MCM-41E formulation was obtained for the *S. pyogenes* ATCC 19615 strain, with the highest inhibition zone diameter of 19 mm at the lowest MIC of 0.5 mg/mL, followed by the *Streptococcus pneumoniae* ATCC 49619 strain (inhibition zone diameter: 17.66 mm; MIC: 1 mg/mL) and then the *S. aureus* ATCC 25923 strain (inhibition zone diameter: 15.66 mm; MIC: 2 mg/mL).

Regarding the Ar@ZnMCM-41 formulation, the best antibacterial effect was also obtained for the *S. pyogenes* ATCC 19615 strain, with the diameter of the inhibition zone being higher (i.e., 21 mm) than the one obtained using the previous formulation at the same MIC of 0.5 mg/mL and an equivalent MBC (i.e., 0.5 mg/mL). Moreover, the Ar@ZnMCM-41 formulation presented an improved antibacterial activity against the *S. aureus* ATCC 25923 strain, with the diameter of the inhibition zone being much higher (i.e., 20.66 mm) at a lower MIC (i.e., 1 mg/mL) than those obtained for the Ar@MCM-41E formulation.

The diameter of the inhibition zone for the *Streptococcus pneumoniae* ATCC 49619 strain was also higher for the Ar@ZnMCM-41 formulation (i.e., 19.66 mm vs. 17.66 mm) at a lower MIC (i.e., 0.5 mg/mL vs. 1 mg/mL).

Both formulations that were tested presented an antimicrobial effect on the *Enterococcus faecalis* ATCC 51299 strain, with a higher inhibition zone diameter being obtained for Ar@ZnMCM-41 (i.e., 16.33 mm vs. 15.33 mm) at a much lower MIC (i.e., 4 mg/mL vs. 8 mg/mL).

It was only possible to test the antimicrobial activity of the two mesoporous silica-type matrices (i.e., MCM-41E and ZnMCM-41) using the disk diffusion method, expressed in millimeters (corresponding to the diameter of the growth of the inhibition zone). Subsequent to the absence of the antimicrobial activity of these compounds in the disk diffusion method, testing of the MIC, MBC, and MFC was no longer necessary.

The two mesoporous silica-type matrices (i.e., MCM-41E and ZnMCM-41) presented much lower antimicrobial activity on the same strains compared to those that had the embedded black chokeberry extract (Table 5).

Hence, based on Table 4 and Table 5, we can conclude that the *Aronia* extract incorporated in the two mesoporous silica-type matrices (i.e., Ar@MCM-41E and Ar@ZnMCM-41) presented antibacterial activity only on Gram-positive bacteria. All of the species of Gram-negative bacteria that were tested were resistant to the action of *Aronia*, as well as the two species of *Candida* that were tested. Moreover, the Ar@ZnMCM-41 sample presented a more potent antibacterial effect than Ar@MCM-41E, probably due to the presence of ZnO nanoparticles, which uniformly decorated the pore walls of silica.3.4. Assessment of the Cytotoxic Effects

**Figure 6:**
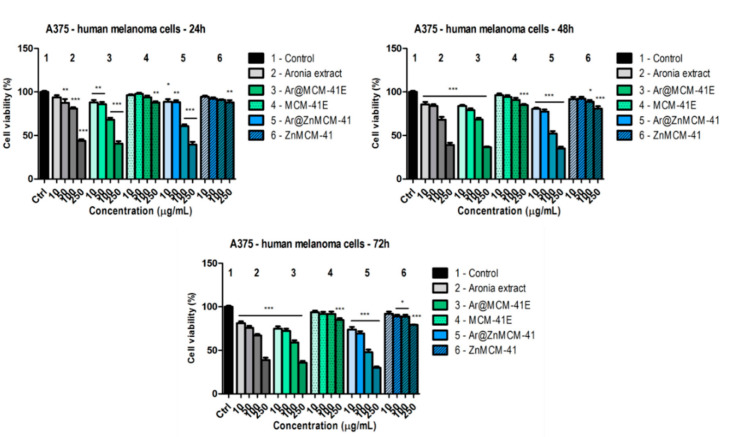
In vitro cytotoxicity evaluation of the *A. melanocarpa* (Michx.) Elliott. Extract (free or encapsulated in the silica-type matrices) and of the MCM-41 silica-type supports (10, 50, 100, and 250 µg/mL) on the A375 human melanoma cell line at 24, 48, and 72 h post-treatment by means of an MTT assay. The results are expressed as cell viability percentage (%) related to the control (i.e., the untreated cells). The data represent the mean values ± standard deviation (SD) of three independent experiments. One-way analysis of variance (ANOVA) followed by a Dunnett’s multiple comparison test was used for comparison among groups (* *p* < 0.05; ** *p* < 0.01; *** *p* < 0.001).

The effects of *A. melanocarpa* (Michx.) Elliott extract, as well as the extract incorporated into the mesoporous silica-type matrices (i.e., Ar@MCM-41E and Ar@ZnMCM-41) and the MCM-41E and ZnMCM-41 matrices, were assessed on a human melanoma cell line (A375) and on a non-tumor cell line (HaCaT human keratinocytes). The samples were evaluated after treatment at different time periods (i.e., 24, 48, and 72 h) and compared to the control (i.e., the untreated cells). Figure 6 depicts the effects of the tested samples on the A375 human melanoma cell line. At 24 h post-treatment, the black chokeberry extract decreased the viability of the tumor cells at the highest doses tested (at 100 µg/mL, cell viability was 80.9 ± 2.4% vs. the control, and, at 250 µg/mL, cell viability was 43.8 ± 3.3% vs. the control). By encapsulating the black chokeberry extract into the mesoporous silica-type matrices, only a slightly higher decrease in the viability of the tumor cells was noticed, indicating that after the 24 h treatment, there were no important differences between the viabilities obtained from the extract alone or in its encapsulated forms. The most significant decrease was observed for the Ar@ZnMCM-41 sample at 250 µg/mL (cell viability was 39.5 ± 5.5% vs. the control), whereas, for Ar@MCM-41E, the viability was 40.7 ± 4.7% vs. the control. The MCM-41E and ZnMCM-41 matrices affected the cell viability to a much lesser extent (at 250 µg/mL, for MCM-41E, cell viability was 87.8 ± 2.5% vs. the control, and, for ZnMCM-41, it was 88 ± 4.3% vs. the control).

At 48 h post-treatment, a dose-dependent decrease in the viability of the melanoma cells was observed for the both black chokeberry extract as well as the black chokeberry extract encapsulated in the matrices. After treatment with the highest concentration (i.e., 250 µg/mL) of black chokeberry extract, the viability of the tumor cells was 39.2 ± 4.1% vs. the control. The highest decrease was obtained in the case of Ar@ZnMCM-41 at 250 µg/mL (cell viability was 35 ± 3.6% vs. the control), followed by Ar@MCM-41E at 250 µg/mL (cell viability was 36.6 ± 1.4% vs. the control). The two matrices also decreased the viability of the cells, especially at the highest dose tested: For MCM-41E, it was 84.7 ± 2.3%, and for ZnMCM-41, it was 80.7 ± 4.9%.

The most significant decrease in the viability of the tumor cells was obtained following treatment with the black chokeberry extract and its encapsulated forms for 72 h. At this time interval, the black chokeberry extract (at 250 µg/mL) provoked a decrease in the viability of the A375 cells to 38.7 ± 4.7% vs. the control. Even at this interval, the most affected were the cells treated with the highest dose of Ar@ZnMCM-41 (for 250 µg/mL, the cell viability was 29.9 ± 2.7% vs. the control), indicating that this encapsulation of the black chokeberry extract in this silica-type matrix could be a promising sample for melanoma treatment; however, additional tests are required to confirm this.

**Figure 7:**
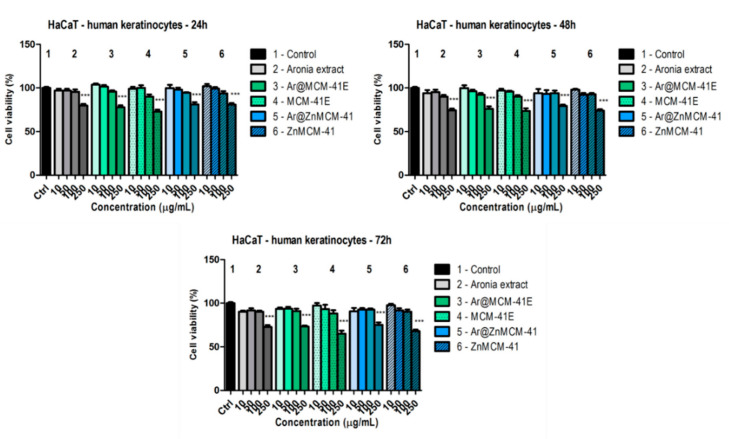
In vitro cytotoxicity evaluation of the *Aronia melanocarpa* (Michx.) Elliott. Extract (alone or encapsulated in MCMs) and of the MCMs (10, 50, 100, and 250 µg/mL) on HaCaT human keratinocytes at 24, 48, and 72 h post-treatment by means of an MTT assay. The results are expressed as cell viability percentage (%) related to the control (i.e., the untreated cells). The data represent the mean values ± SD of three independent experiments. One-way ANOVA followed by a Dunnett’s multiple comparison test was used for comparison among groups (*** *p* < 0.001).

To demonstrate the selectivity on the cancer cell line, the effect of the samples was also determined on a non-tumor cell line. Figure 7 depicts the effect of the aforementioned samples on HaCaT human keratinocytes. Twenty-four hours after treatment, only at the highest dose tested (i.e., 250 µg/mL) could a significant decrease in the viability of the cells compared to control be observed, but the cells were less affected compared to the cancer cells at the same concentration. For cells treated with the black chokeberry extract, compared to the control, the viability was 79.8 ± 3.3%; for Ar@MCM-41E, it was 77.9 ± 3.5%; and, for Ar@ZnMCM-41, it was 81 ± 4.4%. Nevertheless, at 10 and 100 µg/mL, the black chokeberry extract alone or encapsulated in the silica-type matrices elicited a beneficial effect on the HaCaT human keratinocytes, with the viability of the cells being above 94% for all samples. This effect indicates that the extract-loaded supports had good cytocompatibility at doses of up to 100 µg/mL.

At 48 h post-treatment, a significant decrease in the viability of the HaCaT human keratinocytes was noticed at the highest tested dose, i.e., 250 µg/mL. The lowest value was obtained for the black chokeberry extract (cell viability was 74.4 ± 3.1% vs. the control); meanwhile, for Ar@MCM-41E at 250 µg/mL, the viability was 76 ± 4.8% vs. the control, and, for Ar@ZnMCM-41 at the same concentration, it was 79.3 ± 2.7% vs. the control. The MCM-41E carrier provoked, at the highest dose tested, a decrease in cell viability (73.7 ± 5.2%), with the ZnMCM-41 support showing a similar value (74 ± 2.8%).

Following treatment with the samples for 72 h, the previously mentioned effects were maintained, namely, a dose-dependent decrease was noticed, with the most significant results obtained only at the highest dose tested. For the same period of incubation, the melanoma cells were more affected after treatment with the black chokeberry extract alone or with the forms encapsulated in the matrices, indicating that the samples altered the cancer cells to a greater degree than the keratinocytes. At this concentration, the black chokeberry extract decreased cell viability to 72.7 ± 3.6%, whereas for the encapsulated forms, slightly less of a decrease in cell viability was observed (for Ar@MCM-41E, 73.2 ± 2.1% and for Ar@ZnMCM-41, 75.1 ± 4.9% vs. the control). Our results indicate that the black chokeberry extract alone had a slightly more potent effect on the keratinocytes than the encapsulated forms did. Moreover, at the lowest concentrations tested, the encapsulated samples did not significantly affect the viability of the keratinocytes, indicating once more a good cytocompatibility, and, at low doses, they were not toxic toward the HaCaT cells.

For the evaluation of the antimigratory effect of *A. melanocarpa* (Michx.) Elliott extract on A375 human melanoma cells, as well as the black chokeberry extract-loaded mesoporous silica-type matrices (i.e., Ar@MCM-41E and Ar@ZnMCM-41) and the MCM-41E and ZnMCM-41 matrices, a scratch assay was performed. The cells were treated with a concentration of 100 µg/mL from each sample and compared to the control (i.e., untreated cells). This concentration was used for evaluating the antimigratory potential because, at the highest tested concentration, the viability decreased, and the experiment could not be performed.

**Figure 8:**
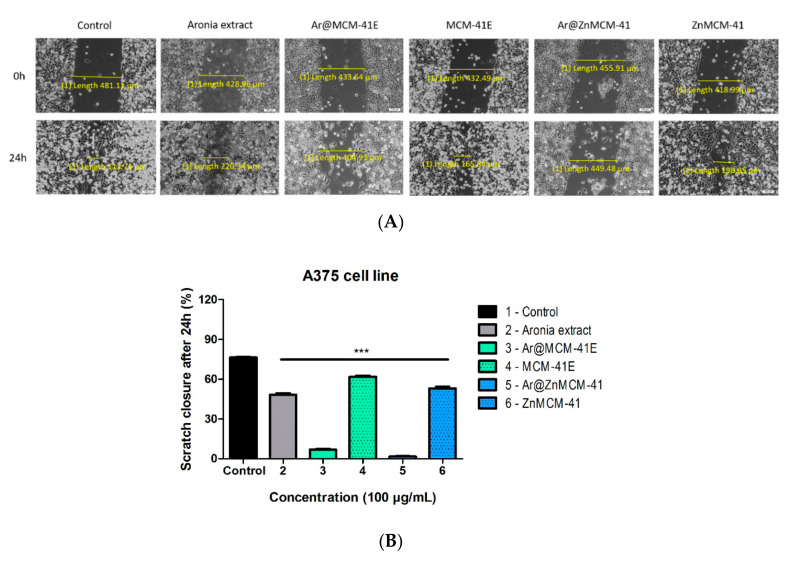
(**A**) In vitro antimigratory potential of the *Aronia melanocarpa* (Michx.) Elliott. Extract (alone or encapsulated in MCMs) and of the MCMs (100 µg/mL) on A375 human melanoma cells. Images were taken by light microscopy at 10× magnification. Pictures were taken at 0 and 24 h post-treatment. The scale bars represent 100 µm. (**B**). The bar graphs are expressed as percentage of scratch closure after 24 h compared to the initial surface. One-way ANOVA followed by a Dunnett’s multiple comparison test was used for comparison among groups (*** *p* < 0.001 vs. the control).

Figure 8 shows that, at the concentration of 100 µg/mL, there was a significant reduction of A375 melanoma cells following treatment with the black chokeberry-loaded silica-type matrices. For Ar@MCM-41E, the scratch closure rate was only 6.8% and, in the case of Ar@ZnMCM-41, the scratch closure rate was even smaller (1.6% vs. the control), indicating an antimigratory effect of the aforementioned samples. Likewise, the black chokeberry extract elicited an antimigratory effect on the cancer cells compared to the control (scratch closure rate of 48.2% for the *Aronia* extract vs. 76.4% for the control). Treatment with the matrices achieved similar results as in the case of the control group.

## 4. Discussion

Nanomedicine is a key science of the 21st century. An increase in research on MSNs as drug carriers for the treatment of various diseases can be observed. MCM-41 is a mesoporous solid, being one of the most widely explored materials for drug delivery [32]. MCM-41 mesoporous silica has an ordered hexagonal pore array of unidirectional and non-interconnecting pores, with diameters of 2–4 nm [33]. The porosity of MCM-41 silica makes it an ideal candidate for the loading and encapsulation of organic molecules or macromolecules such as proteins, DNA, and RNA [34,35,36].

The yield of *Aronia* ethanolic extract was higher (53.8% wt) than that obtained by Rodriguez-Werner et al. for 70% acetonic extracts prepared using ultrasound-assisted extraction (20.6–26.9% wt) [37], probably due to the long process, high temperature, and use of ethanol as solvent for the extraction. It is well known that the conditions of the extraction greatly influence both the yield and the chemical profile of extracts. Our black chokeberry extract had a high polyphenol content (1732 ± 18 mg GAE/100 g), consistent with the amount reported by Jurikova et al. for *Aronia* extracts prepared in different solvents (690–2950 mg GAE/100 g berries) [4]. For a methanolic extract acidified with HCl, a lower total polyphenolic content (10.637 mg/g) and a 10 times higher total anthocyanin content (4.341 mg/g) was obtained, probably due to the addition of acid in the extraction solvent, which favored the extraction of anthocyanins [38]. Other studies reported a lower TPC: 460.5–690.2 mg GAE/100 g for acidified methanolic extracts from *Aronia* Nero [39], 1330 ± 3 mg GAE/100 g for chokeberries from the Czech Republic, or 778–1285 mg GAE/100 g for methanolic extracts from the Aron, Ferbodi, Hugin, Nero, and Viking varieties of black chokeberry [40]. However, an increased amount of TPC was obtained for acidified 80% CH_3_OH extracts, ranging from 1845 to 2340 mg GAE/100 g [41]. Our *Aronia* extract contained a low amount of anthocyanins (0.52 ± 0.02 mg CGE/g), probably because of the extraction temperature (i.e., 80 °C) and the lack of acid in the extraction solvent in comparison to the values reported by Kapci et al. and Tolic et al. for acidified 75% or 95% methanolic extracts—4.5 ± 0.20 mg CGE/ g [42] and 1.41–1.47 mg CGE/g [43], respectively.

Significant RSA of the *Aronia* extract was obtained by the DPPH and ABTS assays, being in the range of 14.06–15.33 mg TE/g, which is higher than the values reported by Kapci et al. (i.e., 11–11.3 mg TE/g) [42] for their extract with a higher amount of anthocyanins.

Concerning the chemical profiling of the black chokeberry extract determined by HPLC–PDA, four standard substances were quantified, namely protocatechuic (62.135 ± 0.005 mg/100 g), chlorogenic acid (141.283 ± 0.045 mg/100 g), caffeic acid (0.725 ± 0.008 mg/100 g), and rutin hydrate (21.773 ± 0.041 mg/100 g), which is consistent with the *Aronia* extract reported by Jurikova et al., who pointed out that chlorogenic acid is the most abundant polyphenolic compound, followed by neochlorogenic and caffeic acids, as well as several flavonoids [4]. For an acidified methanolic extract prepared by ultrasound-assisted extraction, Ochmian et al. reported lower amounts of polyphenols (72–96.6 mg/100 g chlorogenic acid and 3.9–6.1 mg/100 g rutin) [41], while Cebova et al. obtained a higher amount of chlorogenic acid (194.860 ± 0.059 mg/100 g) and lower contents of protocatechuic (34.086 ± 0.770 mg/100 g), caffeic acid (0.478 ± 0.009 mg/100 g), and rutin (1.744 ± 0.000 mg/100 g) than that of our extract [44].

A similar content of chlorogenic acid in the black chokeberry extract was reported by Rop et al. (113.1–1960 mg/100 g) for a methanolic extract obtained at 25 °C [40], while Rodríguez-Werner et al. reported a higher amount (281–301 mg/g) for a 70% acetonic extract prepared by ultrasound-assisted extraction [37].

The nanoconfinement of the black chokeberry polyphenolic extract in the silica-type supports led to obtaining materials with a high content of *Aronia* extract (42–43%) due to the high total pore volume of the supports that preserved the RSA of the extract, as demonstrated by the DPPH assay on solid samples. A higher stability was observed for the Ar@ZnMCM-41 sample, which could explain the better antimicrobial and antitumor activity.

The black chokeberry extract was found to contain very potent antimicrobial compounds. Several studies have depicted the antimicrobial activity of the extracts obtained from this vegetal product. For example, Jurikova et al. concluded that this action could be attributed to several mechanisms and to the synergistic activity of the active compounds presented in black chokeberries, such as anthocyanins, phenolic acids, and weak organic acids [4]. The work performed by Denev et al. highlighted that proanthocyanidins are the major contributors to this effect, as well as to the antioxidant profile of black chokeberries [45]. Epicatechin and quercetin within the extract were found to be the active substances responsible for the antimycotic action on *Candida albicans*, without any effect on *S. aureus* [4,45]. Bräunlich et al. showed the ability of an *A. melanocarpa* L. ethanolic extract to prevent the formation of a biofilm and to inhibit the in vitro growth of *Escherichia* *coli* and *Bacillus* *cereus* [46].

This is the first study that describes the antimicrobial activity of black chokeberry extract embedded in two mesoporous silica-type matrices (i.e., Ar@MCM-41E and Ar@ZnMCM-41), and our findings are consistent with other data from the literature [4,45,47], showing the antimicrobial activity of the black chokeberry extract on Gram-positive bacteria (i.e., *S. aureus* and *B. cereus*) and no antimycotic activity [48]. In 2005, Valcheva-Kuzmanova et al. reported in vitro bacteriostatic activity of the black chokeberry extract on *S. aureus* and *E. coli*. [49]. The two tested samples of black chokeberry showed an antibacterial effect on Gram-positive bacteria with no antimycotic effect on *Candida* spp. From the two studied samples, the black chokeberry extract embedded in mesoporous silica-type matrices modified with ZnO presented the most potent antibacterial activity. An explanation for the lack of effect on Gram-negative bacteria could be that these types of bacteria contain an outer lipopolysaccharide membrane, which blocks the penetration of the hydrophilic substances in the bacterial cells [50]. Moreover, based on the present data, it seems that the embedding of the black chokeberry extract into the mesopores of a ZnO-modified silica-type matrices has a synergistic effect and enhances the antibacterial effect of the black chokeberry extract.

In our study, the greatest inhibition zone diameter (21 mm) was obtained with Ar@ZnMCM-41 on the *S. pyogenes* ATCC19615 strain at the lowest MIC used (i.e., 0.5 mg/mL), followed by the *Streptococcus pneumonieae* ATCC19615 strain (inhibition zone diameter: 19.66 mm; MIC: 0.5 mg/mL) and the *S. aureus* ATCC25923 strain (inhibition zone diameter: 20.66 mm; MIC: 1 mg/mL). In the study of Denev et al., the purified extract containing 20% anthocyanins exhibited the largest inhibition zone diameter of 11 mm for *Proteus vulgaris* G and *S. aureus* ATCC6538P, while the lowest MIC (i.e., 0.156 mg/mL) was found for proanthocyanidins (which was four times lower than that from the purified extract containing 20% anthocyanins) [45]. Although our extract did not present any antimicrobial activity against *Candida* spp., in the study performed by Denev et al., the diameter of the inhibition zone of epicatechin for the *Candida albicans* ATCC10231 strain was 12 mm, with an MIC of 2.5 mg/mL [45]. Differences in the antimicrobial activity of the black chokeberry extract could be attributed to the variation in the concentration of anthocyanins and could be the consequence of the type of extract, solvent used, or cultivation zones of the plant [51].

Regarding other important studies from the scientific literature, a recent study showed that black chokeberry leaf extract also possesses bacteriostatic properties, with *S. aureus*, *Brochothrix thermosphacta*, and *Salmonella enterica* being the most sensitive to the leaf extract compared to *Listeria monocytogenes,* which was found to be the most resistant bacteria to the back chokeberry leaf extract [52]. A recent study published this year by Lee et al. reported that the juice can inhibit the development of an oral streptococcal biofilm at the beginning of its formation by decomposing the formation of extracellular RNA, a structure with a pivotal role in the formation of bacterial biofilms [53]. It seems that the active compounds of black chokeberry (such as flavons, flavonols, flavans, iso/neo-flavonoids, chalcones, and dihydroflavonols) have antibiofilm activity without any toxicity toward the studied species, a beneficial property that lowers the risk of developing resistance, as the current antimicrobials do [4].

In another recent study performed by Brezoiu et al., a moderate improvement in antimicrobial activity was also observed for a polyphenolic extract of *Salvia officinalis* L. when embedded into the mesopores of a silica-type support compared to the free extract [19].

*A. melanocarpa* L. has been previously reported to possess cytotoxic and pro-apoptotic effects on various types of cancer, including colon cancer [2], breast cancer [54], hepatocarcinoma [55], and pancreatic cancer [56]. Our results indicate that the hydroalcoholic extract from black chokeberry elicited a dose- and time-dependent decrease in the viability of melanoma cells. Furthermore, when tested on a non-tumor cell line, namely human keratinocytes, the extract achieved a significant decrease in cell viability only at the highest concentration tested, indicating that the tumor cells were more affected than the keratinocytes. Our data are in accordance with a study performed by Goh et al. [57] that indicated that *A. melanocarpa* L. concentrate did not alter HaCaT cell viability at doses up to 0.3% (*v*/*v*). Bermúdez-Soto et al. investigated the effect of polyphenol-rich chokeberry juice on Caco-2 human colon carcinoma and showed an approximately 70% inhibition in the proliferation of tumor cells and a decrease in the viability of tumor cells to approximately 80% after application of a 5% digested chokeberry juice [58]. In a study performed on melanoma and on normal cell lines, anthocyanin-rich black chokeberry extract showed antiproliferative potential against tumor cells, while having no negative effect on normal cells [59].

In the present study, we evaluated, for the first time, the effect of black chokeberry extract encapsulated into mesoporous silica-type matrices (i.e., MCM-41E and ZnMCM-41) on melanoma and normal cells. Our data show a dose- and time-dependent decrease in the viability of melanoma cells, the most active sample being Ar@ZnMCM-41 at a concentration of 250 µg/mL. On the other hand, the non-tumor cells were less affected by the black chokeberry extract encapsulated in the two matrices. Furthermore, a slight increase in the viability of HaCaT cells was noticed following encapsulation compared to the effect obtained by the black chokeberry extract alone. As reported by Brezoiu et al., an increase in the proliferation of normal cells could be associated with the polyphenolic content of the extract [17]. The effect of the ZnMCM-41 matrix was previously tested on HaCaT cells by Brezoiu et al. The authors reported that, after a 24-h treatment with ZnMCM-41 at a concentration of 100 µg/mL, the mesoporous silica-type matrices modified with ZnO had good cytocompatibility with the keratinocytes [17]. In our study, we obtained similar results—neither the encapsulated samples nor the free matrices significantly altered the viability of HaCaT cells at 10, 50, or 100 µg/mL. In a comprehensive study conducted on resveratrol encapsulated in MSNs, Chaudhary et al. observed that phosphonate-modified MSNs increased the antiproliferative activity of resveratrol, whereas amine-modified MSNs did not alter the proliferation of PC3 prostate cancer cells compared to resveratrol alone [60]. In another study performed on resveratrol encapsulated in colloidal MSNs, it was demonstrated that the encapsulated form enhanced, in a dose-dependent manner, the antiproliferative activity against HT-29 and LS147T colon cancer cell lines as compared to pure resveratrol [61].

The study performed by Gill et al. highlighted the fact that from the three tested extracts of *Aronia* (i.e., *A. arbutifolia*, *A. prunifolia,* and *A. melanocarpa*), the black chokeberry extract had the strongest effect in terms of reducing cell proliferation, having the greatest level of total phenols, individual phenolic acids, and antioxidant activity out of all of the tested samples [62]. In 2014, Thani et al. showed that the in vitro effect of chemotherapy with gemcitabine can be enhanced by the polyphenols from *A. melanocarpa* in the AsPC-1 pancreatic cell line [56]. Moreover, in a study performed by Valcheva-Kuzmanova et al., it was shown that black chokeberry juice can induce a protective in vitro effect based on its antioxidant properties in a model of cisplatin-induced cytotoxicity in the human embryonal kidney cell line HEK293T [63]. In 2018, the group of Do Thi highlighted the antitumor effect of black chokeberry extract on the human liver cancer cell line SK-Hep1. In addition, Choi et al. found, in 2018, that triterpene acid (*3*-*O*-*p*-coumaroyltormentic acid) isolated from black chokeberry powder blocks the formation of breast cancer stem cells by downregulating c-Myc protein, a cancer stem cell survival factor [64].

## 5. Conclusions

This study presented the phytochemical characterization and biological activity evaluation of black chokeberry fruit ethanolic extract obtained from the western part of Romania, both free and loaded into mesoporous MCM-41-type silica (pristine and modified with ZnO nanoparticles). The extract was rich in polyphenols and flavonoids and elicited a significant RSA. HPLC–PDA analysis showed that, among the identified phytochemicals, chlorogenic acid, protocatechuic acid, and rutin hydrate were the most representative. The loading of the black chokeberry extract into mesoporous silica-type supports determined the preservation of its antiradicalic and antimicrobial activity on the tested Gram-positive bacteria. The encapsulation of the *A. melanocarpa* extract into the ZnMCM-41 support led to a slightly better antimicrobial capacity than the free extract due to ZnO synergistic effect. An enhanced antiproliferative effect and antimigratory potential on A375 human melanoma cells, depending on the dose and time, were found when the extract was loaded into mesoporous silica-type matrices. Based on the presented results, one can conclude that mesoporous silica-type matrices could be good hosts for black chokeberry extract. This is a preliminary study that can help to establish new directions to upgrade the use of this new formulation for the studied extract after validation on an increased number of normal and multidrug-resistant strains using experimental animal models of melanoma.

## Figures and Tables

**Table 1:** American Type Culture Collection (ATCC) of the microbial species used.

Type of Microorganism	Microbial Species	ATCC
Gram-negative bacilli	*Salmonella enterica* serotip *typhimurium*	14028
	*Shigella flexneri* serotip 2b	12022
	*Escherichia coli*	25922
	*Pseudomonas aeruginosa*	27853
Gram-positive cocci	*Enterococcus faecalis*	51299
	*Staphylococcus aureus*	25923
	*Streptococcus pneumoniae*	49619
	*Streptococcus pyogenes*	19615
Fungi	*Candida albicans*	10231
	*Candida parapsilosis*	22019

**Table 2:** Total polyphenolic content (TPC), total flavonoid content (TFC), total anthocyanin content (TAC), IC_50%_ value (50% of the DPPH free radical inhibition), and radical scavenging activity (RSA) by the DPPH and ABTS methods, as well as the identification and quantification of polyphenolic compounds by reverse-phase high-performance liquid chromatography with a photodiode array detector (HPLC–PDA) for the *Aronia* extract.

Extract	Yield (%)	TPC (mg GAE/g)	TFC (mg RE/g)	TAC (mg CGE/g)	IC_50%_ (mg extract/mL)	RSA DPPH (mg TE/g)	RSA ABTS (mg TE/g)
*A. melano-carpa*	53.8	17.32 ± 0.18	6.98 ± 0.28	0.52 ± 0.02	11.92	14.06 ± 1.01	15.33 ± 0.42
HPLC–PDA composition							
Standard substances		Protocatechuic acid		Chlorogenic acid		Caffeic acid	Rutin hydrate
Concentration in extract (mg/100 g)		62.135 ± 0.005		141.283 ± 0.045		0.725 ± 0.008	21.773 ± 0.041

All values are expressed as per gram of dried berries. GAE, gallic acid equivalent; RE, rutin hydrate equivalent; CGE, cyanidin-3-glucoside equivalent; TE, Trolox equivalent.

**Table 3:** Textural parameters of the supports and the corresponding *Aronia*-loaded supports.

Support Type	Support				Ar@support			
	ZnO	d_BJH_ (nm)	S_BET_ (m^2^/g)	V_pore_ (cm^3^/g)	Extract (% wt)	d_BJH_ (nm)	S_BET_ (m^2^/g)	V_pore_ (cm^3^/g)
	(% wt)							
MCM-41E	-	2.81	781	0.78	43	2.52	89	0.12
ZnMCM-41	12	2.66	620	0.8	42	-	17	0.11

d_BJH_, average pore size determined by the Barrett–Joyner–Halenda method; S_BET_, specific surface area computed by the Brunauer–Emmett–Teller method; V_pore_, total pore volume.

**Table 4:** The diameter of inhibition growth zone, the minimum inhibitory concentration, and minimum bactericidal concentration of the black chokeberry extract free and embedded in two mesoporous silica-type matrices (i.e., Ar@MCM-41E and Ar@ZnMCM-41) for the tested strains.

Microbial Species	*Aronia*			Ar@MCM-41E			Ar@ZnMCM-41		
	Disk Diffusion Method	Dilution Method		Disk Diffusion Method	Dilution Method		Disk Diffusion	Dilution Method	
	Inhibition Zone Diameter	MIC	MFC MBC	Inhibition Zone Diameter	MIC	MFC MBC	Inhibition Zone Diameter	MIC	MFC MBC
	(mm)	(mg/mL)		(mm)	(mg/mL)		(mm)	(mg/mL)	
*Salmonella enterica*	7.66	**-**	-	9.66	-	-	11.66	-	-
*Shigella flexneri* serotip 2b	7.33	-	-	8.33	-	-	11.33	-	-
*Escherichia coli*	8.66	-	-	9.66	-	-	11.66	-	-
*Pseudomonas aeruginosa*	7	-	-	7.33	-	-	8.33	-	-
*Enterococcus faecalis*	12.66	-	-	15.33	8	8	16.33	4	8
*Staphylococcus aureus*	15	2	2	15.66	2	2	20.66	1	2
*Streptococcus pneumoniae*	15.33	2	2	17.66	1	1	19.66	0.5	1
*Streptococcus pyogenes*	16.66	1	1	19	0.5	1	21	0.5	0.5
*Candida albicans*	7	-	-	7	-	-	7.66	-	-
*Candida parapsilosis*	7	-	-	7.33	-	-	7.33	-	-

MIC, minimum inhibitory concentration; MBC, minimum bactericidal concentration; MFC, minimum fungicidal concentration.

**Table 5:** The antimicrobial activity of the two mesoporous silica-type matrices using the disk diffusion method (MCM-41E and ZnMCM-41).

Microbial Species	MCM-41E	ZnMCM-41
	Inhibition Zone Diameter (mm)	
*Salmonella enterica*	7	10.66
*Shigella flexneri* serotip 2b	7	10.66
*Escherichia coli*	7	10.66
*Pseudomonas aeruginosa*	7	10.66
*Enterococcus faecalis*	7	7.33
*Staphylococcus aureus*	8	10.66
*Streptococcus pneumoniae*	9	10.66
*Streptococcus pyogenes*	9.33	11.33
*Candida albicans*	8.33	9.66
*Candida parapsilosis*	7.66	9.33

## References

[1] Buda V., Andor M., Diana A., Ardelean F., Pavel I.Z., Dehelean C., Soica C., Folescu R., Andrei F., Danciu C. (2020). Cardioprotective Effects. Natural Products-From Bioactive Molecules to Human Health.

[2] Kulling S.E., Rawel H. (2008). Chokeberry (Aronia melanocarpa)—A Review on the Characteristic Components and Potential Health Effects. Planta Med..

[3] Kokotkiewicz A., Jaremicz Z., Luczkiewicz M. (2010). AroniaPlants: A Review of Traditional Use, Biological Activities, and Perspectives for Modern Medicine. J. Med. Food.

[4] Jurikova T., Mlcek J., Škrovánková S., Sumczynski D., Sochor J., Hlavacova I., Snopek L., Orsavová J. (2017). Fruits of Black Chokeberry Aronia melanocarpa in the Prevention of Chronic Diseases. Molecules.

[5] Brand M.H., Connolly B.A., Levine L.H., Richards J.T., Shine S.M., Spencer L.E. (2017). Anthocyanins, total phenolics, ORAC and moisture content of wild and cultivated dark-fruited Aronia species. Sci. Hortic..

[6] Serna-Guerrero R., Sayari A. (2007). Applications of Pore-Expanded Mesoporous Silica. 7. Adsorption of Volatile Organic Compounds. Environ. Sci. Technol..

[7] Mitran R.-A., Deaconu M., Matei C., Berger D. (2019). Mesoporous Silica as Carrier for Drug-Delivery Systems. Nanocarriers Drug Deliv..

[8] Mendiratta S., Hussein M., Nasser H.A., Ali A.A.A. (2019). Multidisciplinary Role of Mesoporous Silica Nanoparticles in Brain Regeneration and Cancers: From Crossing the Blood–Brain Barrier to Treatment. Part. Part. Syst. Charact..

[9] Gharpure K.M., Wu S.Y., Li C., Lopez-Berestein G., Sood A.K. (2015). Nanotechnology: Future of Oncotherapy. Clin. Cancer Res..

[10] Koninti R.K., Palvai S., Satpathi S., Basu S., Hazra P. (2016). Loading of an anti-cancer drug into mesoporous silica nano-channels and its subsequent release to DNA. Nanoscale.

[11] Paris J.L., Baeza A., Vallet-Regí M. (2019). Overcoming the stability, toxicity, and biodegradation challenges of tumor stimuli-responsive inorganic nanoparticles for delivery of cancer therapeutics. Expert Opin. Drug Deliv..

[12] Bollu V.S., Barui A.K., Mondal S.K., Prashar S., Fajardo M., Briones D., Rodriguez-Dieguez A., Patra C.R., Gómez-Ruiz S. (2016). Curcumin-loaded silica-based mesoporous materials: Synthesis, characterization and cytotoxic properties against cancer cells. Mater. Sci. Eng. C.

[13] Deaconu M., Brezoiu A.-M., Mitran R.-A., Nicu I., Manolescu B., Matei C., Berger D. (2020). Exploiting the zwitterionic properties of lomefloxacin to tailor its delivery from functionalized MCM-41 silica. Microporous Mesoporous Mater..

[14] Georgescu D., Brezoiu A.-M., Mitran R.-A., Berger D., Matei C., Negreanu-Pirjol B. (2017). Mesostructured silica–titania composites for improved oxytetracycline delivery systems. C. R. Chim..

[15] Deaconu M., Pintilie L., Vasile E., Mitran R.-A., Pircalabioru G.G., Matei C., Chifiriuc M.C., Berger D. (2019). Norfloxacin delivery systems based on MCM-type silica carriers designed for the treatment of severe infections. Mater. Chem. Phys..

[16] Tzankova V., Aluani D., Yordanov Y., Valoti M., Frosini M., Spassova I., Kovacheva D., Tzankov B. (2019). In vitro toxicity evaluation of lomefloxacin-loaded MCM-41 mesoporous silica nanoparticles. Drug Chem. Toxicol..

[17] Brezoiu A.-M., Matei C., Deaconu M., Stanciuc A.-M., Trifan A., Gaspar-Pintiliescu A., Berger D. (2019). Polyphenols extract from grape pomace. Characterization and valorisation through encapsulation into mesoporous silica-type matrices. Food Chem. Toxicol..

[18] Brezoiu A.-M., Bajenaru L., Berger D., Mitran R.-A., Deaconu M., Lincu D., Stoica Guzun A., Matei C., Moisescu M.G., Negreanu-Pirjol T. (2020). Effect of Nanoconfinement of Polyphenolic Extract from Grape Pomace into Functionalized Mesoporous Silica on Its Biocompatibility and Radical Scavenging Activity. Antioxidants.

[19] Brezoiu A.-M., Prundeanu M., Berger D., Deaconu M., Matei C., Oprea O., Vasile E., Negreanu-Pirjol T., Muntean D., Danciu C. (2020). Properties of Salvia officinalis L. and Thymus serpyllum L. Extracts Free and Embedded into Mesopores of Silica and Titania Nanomaterials. Nanomaterials.

[20] Ciobanu M., Parvulescu V., Paun G., Savin S., Albu B., Munteanu C., Cusu J.P., Atkinson I., Culita D.C., Petcu G. (2019). Development of a new (bio)hybrid matrix based on Althaea officinalis and Betonica officinalis extracts loaded into mesoporous silica nanoparticles for bioactive compounds with therapeutic applications. J. Drug Deliv. Sci. Technol..

[21] Ćujić-Nikolić N., Stanisavljević N., Šavikin K., Kalušević A., Nedović V., Samardžić J., Janković T. (2019). Chokeberry polyphenols preservation using spray drying: Effect of encapsulation using maltodextrin and skimmed milk on their recovery following in vitro digestion. J. Microencapsul..

[22] Ćujić-Nikolić N., Trifković K.T., Bugarski B., Ibric S., Pljevljakusic D., Šavikin K. (2016). Chokeberry (Aronia melanocarpa L.) extract loaded in alginate and alginate/inulin system. Ind. Crops Prod..

[23] Kordsmeier M., Howard L. (2011). Storage effects of gel encapsulation on stability of chokeberry monomeric anthocyanins, procyanidins, color density, and percent polymeric color. Discov. Stud. J. Dale Bumpers Coll. Agric. Food Life Sci..

[24] Janiszewska-Turak E., Sak A., Witrowa-Rajchert D. (2019). Influence of the carrier material on the stability of chokeberry juice microcapsules. Int. Agrophys..

[25] Brezoiu A.-M., Deaconu M., Nicu I., Vasile E., Mitran R.-A., Matei C., Berger D. (2019). Heteroatom modified MCM-41-silica carriers for Lomefloxacin delivery systems. Microporous Mesoporous Mater..

[26] Cockerill F., Patel J., Alder J., Bradford P., Dudley M., Eliopoulos G. (2013). Performance Standards for Antimicrobial Susceptibility Testing 2013.

[27] Arendrup M.C., Hope W., Cuenca-Estrella M., Lass-Flörl C. (2012). EUCAST technical note on the EUCAST definitive document EDef 7.2: Method for the determination of broth dilution minimum inhibitory concentrations of antifungal agents for yeasts EDef 7.2 (EUCAST-AFST)*. Clin. Microbiol. Infect..

[28] Muntean D., Licker M., Alexa E., Popescu I., Jianu C., Buda V., Dehelean C.A., Ghiulai R., Horhat F.G., Horhat D. (2019). Evaluation of essential oil obtained from Mentha×piperita L. against multidrug-resistant strains. Infect. Drug Resist..

[29] Danciu C., Bojin F., Ambrus R., Muntean D., Soica C., Paunescu V., Cristea M., Pinzaru I., Dehelean C. (2017). Physico-chemical and Biological Evaluation of Flavonols: Fisetin, Quercetin and Kaempferol Alone and Incorporated in beta Cyclodextrins. Anti-Cancer Agents Med. Chem..

[30] Danciu C., Muntean D., Alexa E., Watz C., Oprean C., Zupkó I., Bor A., Minda D., Proks M., Buda V. (2018). Phytochemical Characterization and Evaluation of the Antimicrobial, Antiproliferative and Pro-Apoptotic Potential of Ephedra alata Decne. Hydroalcoholic Extract against the MCF-7 Breast Cancer Cell Line. Molecules.

[31] Moacă E.-A., Pavel I.Z., Danciu C., Crainiceanu Z., Minda D., Ardelean F., Antal D.S., Ghiulai R., Cioca A., Derban M. (2019). Romanian Wormwood (*Artemisia absinthium* L.): Physicochemical and Nutraceutical Screening. Molecules.

[32] Narayan R., Nayak U.Y., Raichur A.M., Garg S. (2018). Mesoporous Silica Nanoparticles: A Comprehensive Review on Synthesis and Recent Advances. Pharmaceutics.

[33] Bhattacharyya S., Lelong G., Saboungi M.-L. (2006). Recent progress in the synthesis and selected applications of MCM-41: A short review. J. Exp. Nanosci..

[34] Deodhar G.V., Adams M.L., Trewyn B.G. (2016). Controlled release and intracellular protein delivery from mesoporous silica nanoparticles. Biotechnol. J..

[35] Cha W., Fan R., Miao Y., Zhou Y., Qin C., Shan X., Wan X., Li J. (2017). Mesoporous Silica Nanoparticles as Carriers for Intracellular Delivery of Nucleic Acids and Subsequent Therapeutic Applications. Molecules.

[36] Moeller K., Müller K., Engelke H., Bräuchle C., Wagner E., Bein T. (2016). Highly efficient siRNA delivery from core–shell mesoporous silica nanoparticles with multifunctional polymer caps. Nanoscale.

[37] Rodríguez-Werner M., Winterhalter P., Esatbeyoglu T. (2019). Phenolic Composition, Radical Scavenging Activity and an Approach for Authentication of Aronia melanocarpa Berries, Juice, and Pomace. J. Food Sci..

[38] Jakobek L., Šeruga M., Medvidović-Kosanović M., Novak I. (2007). Antioxidant Activity and Polyphenols of Aronia in Comparison to other Berry Species. Agric. Conspec.Sci..

[39] Benvenuti S., Pellati F., Melegari M., Bertelli D. (2006). Polyphenols, Anthocyanins, Ascorbic Acid, and Radical Scavenging Activity of Rubus, Ribes, and Aronia. J. Food Sci..

[40] Rop O., Micek J., Jurikova T., Valsikova M., Sochor J., Reznicek V., Kramarova D. (2010). Phenolic content, antioxidant capacity, radical oxygen species scavenging and lipid peroxidation inhibiting activities of extracts of five black chokeberry (Aronia melanocarpa (Michx.) Elliot) cultivars. J. Med. Plants Res..

[41] Ochmian I.D., Grajkowski J., Smolik M. (2012). Comparison of Some Morphological Features, Quality and Chemical Content of Four Cultivars of Chokeberry Fruits (Aronia melanocarpa). Not. Bot. Horti Agrobot. Cluj-Napoca.

[42] Kapci B., Neradová E., Čížková H., Voldřich M., Rajchl A., Capanoglu E. (2013). Investigating the antioxidant potential of chokeberry (Aronia melanocarpa) products. J. Food Nut. Res..

[43] Tolić M.-T., Dubrava A.G., Šuška C.H., Jurčević I.L., Krbavčić I.P., Marković K., Vahčić N. (2015). Phenolic Content, Antioxidant Capacity and Quality of Chokeberry (Aronia Melanocarpa) Products. Food Technol. Biotechnol..

[44] Cebová M., Klimentova J., Janega P., Pechanova O. (2017). Effect of Bioactive Compound of Aronia melanocarpa on Cardiovascular System in Experimental Hypertension. Oxid. Med. Cell. Longev..

[45] Denev P., Číž M., Kratchanova M., Blazheva D. (2019). Black chokeberry (Aronia melanocarpa) polyphenols reveal different antioxidant, antimicrobial and neutrophil-modulating activities. Food Chem..

[46] Bräunlich M., Økstad O.A., Slimestad R., Wangensteen H., Malterud K.E., Barsett H. (2013). Effects of Aronia melanocarpa Constituents on Biofilm Formation of Escherichia coli and Bacillus cereus. Molecules.

[47] Denev P., Kratchanova M., Ciz M., Lojek A., Vasicek O., Blazheva D., Nedelcheva P., Vojtek L., Hyršl P. (2014). Antioxidant, antimicrobial and neutrophil-modulating activities of herb extracts. Acta Biochim. Pol..

[48] Liepiņa I., Nikolajeva V., Jākobsone I. (2013). Antimicrobial activity of extracts from fruits of Aronia melanocarpa and Sorbus aucuparia. Environ. Exp. Biol..

[49] Valcheva-Kuzmanova S.V., Belcheva A. (2006). Current knowledge of Aronia melanocarpa as a medicinal plant. Folia Med..

[50] Raudsepp P., Koskar J., Anton D., Meremäe K., Kapp K., Laurson P., Bleive U., Kaldmäe H., Roasto M., Püssa T. (2018). Antibacterial and antioxidative properties of different parts of garden rhubarb, blackcurrant, chokeberry and blue honeysuckle. J. Sci. Food Agric..

[51] Dorneanu R., Cioanca O., Chifiriuc O., Albu E., Tuchiluş C., Mircea C., Salamon I., Hăncianu M. (2017). Synergic benefits of Aronia melanocarpa anthocyanin-rich extracts and antibiotics used for urinary tract infections. Farmacia.

[52] Efenberger-Szmechtyk M., Nowak A., Czyzowska A., Kucharska A.Z., Fecka I. (2020). Composition and Antibacterial Activity of Aronia melanocarpa (Michx.) Elliot, Cornus mas L. and Chaenomeles superba Lindl. Leaf Extracts. Molecules.

[53] Lee H.-J., Oh S.Y., Hong S.-H. (2020). Inhibition of streptococcal biofilm formation by Aronia by extracellular RNA degradation. J. Sci. Food Agric..

[54] Kedzierska M., Malinowska J., Kontek B., Kolodziejczyk-Czepas J., Czernek U., Potemski P., Piekarski J.H., Jeziorski A., Olas B. (2013). Chemotherapy modulates the biological activity of breast cancer patients plasma: The protective properties of black chokeberry extract. Food Chem. Toxicol..

[55] Gao N., Wang Y., Jiao X., Chou S., Li E., Li B. (2018). Preparative Purification of Polyphenols from Aronia melanocarpa (Chokeberry) with Cellular Antioxidant and Antiproliferative Activity. Molecules.

[56] Thani N.A.A., Keshavarz S.A., Lwaleed B., Cooper A.J., Rooprai H.K. (2014). Cytotoxicity of gemcitabine enhanced by polyphenolics from Aronia melanocarpa in pancreatic cancer cell line AsPC-1. J. Clin. Pathol..

[57] Goh A.R., Youn G.S., Yoo K.Y., Won M.H., Han S.-Z., Lim S.S., Lee K.-W., Choi S.Y., Park J. (2016). Aronia melanocarpa Concentrate Ameliorates Pro-Inflammatory Responses in HaCaT Keratinocytes and 12-O-Tetradecanoylphorbol-13-Acetate-Induced Ear Edema in Mice. J. Med. Food.

[58] Bermúdez-Soto M.J., Larrosa M., García-Cantalejo J.M., Espín J.C., Tomás-Barberán F.A., García-Conesa M.T. (2007). Up-regulation of tumor suppressor carcinoembryonic antigen-related cell adhesion molecule 1 in human colon cancer Caco-2 cells following repetitive exposure to dietary levels of a polyphenol-rich chokeberry juice. J. Nutr. Biochem..

[59] Diaconeasa Z., Ayvaz H., Ruginǎ D., Leopold L., Stǎnilǎ A., Socaciu C., Tăbăran F., Luput L., Mada D.C., Pintea A. (2017). Melanoma Inhibition by Anthocyanins Is Associated with the Reduction of Oxidative Stress Biomarkers and Changes in Mitochondrial Membrane Potential. Plant Foods Hum. Nutr..

[60] Chaudhary Z., Subramaniam S., Khan G.M., Abeer M.M., Qu Z., Janjua T., Kumeria T., Batra J., Popat A. (2019). Encapsulation and Controlled Release of Resveratrol Within Functionalized Mesoporous Silica Nanoparticles for Prostate Cancer Therapy. Front. Bioeng. Biotechnol..

[61] Summerlin N., Qu Z., Pujara N., Sheng Y., Jambhrunkar S., McGuckin M.A., Popat A. (2016). Colloidal mesoporous silica nanoparticles enhance the biological activity of resveratrol. Colloids Surf. B Biointerfaces.

[62] Gill N.K., Rios D., Osorio-Camacena E., Mojica B.E., Kaur B., Soderstrom M.A., Gonzalez M., Plaat B., Poblete C., Kaur N. (2020). Anticancer Effects of Extracts from Three Different Chokeberry Species. Nutr. Cancer.

[63] Valcheva-Kuzmanova S., Beronova A.B., Momekov G. (2013). Protective effect of aronia melanocarpa fruit juice in a model of cisplatin-induced cytotoxicity in vitro. Folia Med..

[64] Choi H.S., Kim S.-L., Kim J.-H., Deng H.-Y., Yun B.-S., Lee D.-S. (2018). Triterpene Acid (3-O-p-Coumaroyltormentic Acid) Isolated from Aronia Extracts Inhibits Breast Cancer Stem Cell Formation through Downregulation of c-Myc Protein. Int. J. Mol. Sci..

